# Effects of High Carbohydrate vs. High Protein Pre-exercise Feedings on Psychophysiological Responses to High Intensity Interval Exercise in Overweight Perimenopausal Women

**DOI:** 10.3389/fnut.2018.00141

**Published:** 2019-01-22

**Authors:** Maria Kotopoulea-Nikolaidi, Emily Watkins, Ifigeneia Giannopoulou

**Affiliations:** School of Sport and Service Management, University of Brighton, Eastbourne, United Kingdom

**Keywords:** perimenopausal, high intensity interval exercise, carbohydrates, mood, cognitive function

## Abstract

**Objective:** To investigate (a) the acute effect of a bout of high intensity interval exercise (HIIE) on mood, cognitive function, and blood pressure in overweight perimenopausal women and (b) to compare the effects of high carbohydrate vs. high protein pre-HIIE feedings on exercise capacity, mood and cognitive function in this population.

**Methods:** Twelve, overweight, perimenopausal women (age; 45.5 ± 2.3 years, body fat; 32.2 ± 2.1%) completed a bout of HIIE under 3 pre-exercise feedings (HCHO: high-carbohydrate-low-protein; LCHP: low-carbohydrate-high-protein; control: fasted) in a randomized crossover design. HIIE consisted of 4 intervals of 4 min walking at 85–90% of maximum heart rate and 3 min recovery. Before and after HIIE, the shortened version of the profile of mood state questionnaire, the exercise—induced feeling inventory questionnaire and three cognitive function tests (Stroop test, Shift Stroop test, *n*-back test) were administered. Blood pressure was measured pre- and post-exercise. Following HIIE a performance test to volitional fatigue was conducted.

**Results:** A single bout of HIIE resulted in significant reductions in blood pressure and improvements in cognitive capacity (*p* < 0.05). Both the HCHO and LCHP feedings led to significantly longer exercise performance compared to CON (422 ± 71 s and 340 ± 46 vs. 240 ± 32 s, respectively, *p* < 0.01), with a 1.22-fold greater increase in performance time in HCHO compared to LCHP, although not statistically significant (*p* > 0.05). Only the HCHO trial improved total mood disturbance and positive engagement 1 h-post-exercise compared to CON (*p* < 0.05). HCHO and LCHP improved physical exhaustion and revitalization feelings post-exercise vs. CON (*p* < 0.01).

**Conclusions:** A single HIIE session improves cognitive function and blood pressure in overweight perimenopausal women. High-carbohydrate pre-HIIE feedings can result in greater enhancements in mood and positive engagement to exercise and may improve exercise performance compared to a high-protein meal.

## Introduction

Menopausal transition is a biological phenomenon that can change the endocrine stability and profile (1). During the peri- and postmenopausal years, the reduction of estrogen levels leads to sarcopenia, accumulation of body fat, and cardiometabolic disturbances such as hypertension and arteriosclerosis (2). These conditions, in combination with the observed reduction in regular exercise participation, further increase the risk of obesity and related chronic diseases (2). Moreover, significant disturbances in mood state such as anxiety and depression and reductions in cognitive function have been reported, increasing the risk of morbidity and mortality (1).

Regular physical activity is an effective treatment to drive cardiovascular and metabolic improvements and increase exercise capacity in older post-menopausal women (3). Continuous exercise even in the form of a single bout, has been shown to lead to considerable psychophysiological benefits such as reduced blood pressure, improved mood, and cognitive function in this population (4). Recently, high-intensity interval exercise (HIIE) has been reported to be an effective mode of exercise with similar or greater effects on exercise capacity, cardiovascular and metabolic health in older clinical populations. However, there is a paucity of research on the effects of interval exercise on the mood state, cognition and cardiovascular risk factors such as blood pressure on peri- and postmenopausal women (5).

The combination of diet and exercise has been established as the optimal lifestyle treatment for aging and chronic diseases as demonstrated by their additive beneficial effects on exercise capacity, body composition, mood, and cognitive state (6). In the past, high carbohydrate diets were traditionally prescribed to older and/or obese women, with improvements reported in glucose metabolism, body composition, hypertension, mood, and cognition, particularly when combined with exercise (7). Currently, there is an emphasis on high-protein diets with the majority of studies reporting significant nutritional and cardiometabolic benefits in clinical populations, while on the other hand some studies demonstrating adverse effects and paucity of data on mood and cognition in older women due to the lower carbohydrate availability in the brain (8–10). Hence, it is still not clear what the optimal nutritional and exercise treatment plan for older women is. Furthermore, there is lack of research on the effects that a high protein, low carbohydrate pre-exercise meal might have on exercise capacity and mood and cognitive responses to a bout of interval exercise in this high risk, understudied population. Numerous research studies have established the importance of high carbohydrate pre-exercise feedings on exercise capacity, delay of fatigue and improved mental health, primarily due to an increased glucose availability for the musculature and the brain (11, 12). However, no studies have investigated whether the low carbohydrate availability of high protein diets and pre-exercise meals such as breakfast, will adversely affect the positive effects of exercise on mood and cognition of older women. Furthermore, no research data have been reported on the effects of such low carbohydrate meals on enjoyment of exercise and feelings of exhaustion, two major contributors of exercise adherence in this population. This is even more important to study in the high intensity interval type of workouts that are currently prescribed in older populations and predominantly rely on high carbohydrate availability for energy production and optimum exercise training adaptations (13, 14).

The aim of the present study was 2-fold. Firstly, to investigate the acute effects of HIIE on mood, cognitive function and blood pressure in overweight, perimenopausal women. Secondly, to investigate whether manipulation of carbohydrate and protein availability pre-exercise, can alter exercise tolerance and mood, cognitive function, and blood pressure responses in this population.

## Materials and Methods

### Participants

Twelve perimenopausal women (40–50 years old) volunteered to participate in this study. All women were recreationally active (<3 days/week), healthy, non-smokers and had not taken any medication for the last 12 months. Women provided written informed consent before participation in this study. At their initial visit women completed a medical questionnaire and a menstrual history questionnaire. Procedures were approved by the University of Brighton ethics committee and were conformed to the standards set by the Declaration of Helsinki.

### Experimental Design and Measurements

Participants reported to the laboratory on five separate occasions. All participants completed a preliminary testing session, a familiarization session and three experimental trials in a randomized crossover design separated by a minimum of 3 days. Each experimental trial was completed the same time of the day, under the same environmental conditions, with the same people present in the laboratory (the experimenter and a lab assistant) in order to reduce the confounding effects of the socialization aspect of exercise. The experimental trials were completed during the follicular phase of the menstrual cycle.

In visit 1, participants' measurements of height, body mass (Detecto, Missouri, USA) and body composition were conducted (BodPod COSMED Srl, Surrey, UK). Peak oxygen uptake (VO_2peak_) was assessed using a continuous incremental exercise test to exhaustion on a treadmill (Life Fitness, UK, Cambridgeshire, UK) (13). Pulmonary gases via Douglas bags (Type 543, Georg Fischer AG, Schaffhausen, Switzerland), heart rate (HR) (Polar Electro, Kempele, Finland) and rating of perceived exertion (RPE) were measured at the last minute of each stage. The 2nd visit consisted of a familiarization session, to ensure that the correct speed and inclination of the treadmill were identified from the VO_2peak_ test to achieve the 85–90% of HRmax for the HIIE protocol and the 100% of HRmax for the performance test. Participants were also familiarized with the cognitive function tasks.

In the experimental trial visits, participants arrived at the laboratory at 9:00 a.m. after having fasted overnight from 10 p.m. In order to control pre-exercise nutritional status, each subject completed a 2-day dietary recall at their initial visit and replicated this in the 48 h prior to the experimental trials. Three hours prior to the exercise test, participants ate the prescribed breakfast or no breakfast (control) in a randomized order. The breakfasts were isocaloric (500 ± 15 kcal) and consisted of a high-carbohydrate (HCHO) (60% carbohydrates, 25% fat, 14% protein) vs. a low-carbohydrate-high-protein feeding (LCHP) (40% carbohydrates, 25% fat, 35% protein). Thirty minutes before the exercise test, participants were randomly provided with a HCHO or LCHP sports drink or water (control). In the HCHO condition participants consumed 5 ml.kg^−1^ of body mass of a commercially available sports drink (Lucozade-6.4% carbohydrates) whereas in the LCHP condition participants consumed 0.25 gr.kg^−1^ of body mass pure whey protein (Bulk Powders, UK) diluted in 5 ml.kg^−1^ of body mass water (15). In the control condition participants consumed 5 ml.kg^−1^ of body mass water. The beverages in the main experimental conditions were isovolumic and isocaloric (HCHO; 310 ± 30 ml, 87 ± 10 kcal vs. LCHP; 310 ± 30 ml, 88 ± 11 kcal). Participants were blinded to the content of the beverages. Following drink consumption, participants undertook the HIIE protocol and then a physical performance test.

**Figure 1:**
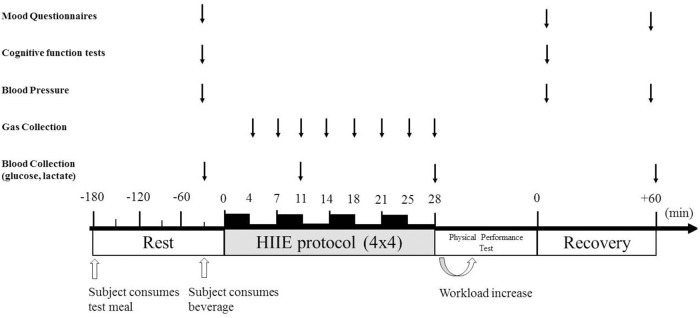
Experimental protocol. Protocol timeline illustrates mood state questionnaires, cognitive function tests, blood pressure collection, gas collection, blood collection, exercise periods, and timing of ingesting high-carbohydrate and high-protein meal or fasted condition before exercise and either isotonic beverage, whey protein beverage or water 30 min before exercise.

The high-intensity interval exercise (HIIE) protocol consisted of a 10-min self-paced warm-up followed by walking or jogging uphill for 4-min intervals (4 × 4) at 85–90% of HRmax (13) (Figure 1). Each interval was separated by a 3-min active recovery, walking at 50–60% of HRmax. At the last minute of each 4-min interval and 3-min recovery, an expired air gas sample, HR and RPE were measured. Prior, post and 1 h-post-exercise, resting blood pressure was measured by an electronic blood pressure monitor (Boso Medicus, Bosch + SoHn, Jungingen, Germany) after 10 min of supine rest (16). The average of 3 measurements was recorded. Capillary blood samples were collected by fingerpick sampling from the right hand pre, during and post-exercise for lactate and glucose analysis using the YSI 2300 (Stat Plus, Hampshire, UK). At completion of the HIIE, a time to volitional fatigue physical performance test was performed on a treadmill at 100% of HR max. Pilot data on five participants, revealed a good level of reliability for the physical performance test (Interclass Correlation Coefficient = 0.877, *p* < 0.05).

Before and after the completion of the exercise test, three cognitive function tests were administered to assess specific executive functions: inhibition of prepotent responses (Stroop test), shifting of mental sets (Shift Stroop test), and monitoring and updating of working memory representations (*n*-back test) (17), These three tests are measuring different areas of the frontal lobe and provide a comprehensive assessment of cognitive function (17). In the Stroop test participants were presented via laptop with a word that named a color and were asked to respond as quickly as possible and choose the color of the word and not the actual word. The Shift Stroop test included two tasks which were switching and participants were asked to react as soon as possible to the tasks. In the *n*-Back test participants were presented via laptop with a sequence of letters and the task consisted of indicating when the current letter matched the one from three letters earlier in the sequence.

Mood was assessed pre, post and 1-h post-exercise using the shortened version of the Profile of Mood States (POMS) questionnaire and the Exercise—Induced Feeling Inventory (EIFI) questionnaire (18, 19). The modified POMS questionnaire consisted of 24-item mood adjective checklist assessing six subscales (anger, confusion, depression, fatigue, tension, and vigor) that were combined to form the overall measure of total mood disturbance (TMD). The EIFI questionnaire consisted of four distinct subscales including positive engagement, revitalization, tranquility, and physical exhaustion.

### Statistical Analysis

Data were analyzed using SPSS 21.0. A two-way (condition × time) repeated measures analysis of variance (ANOVA) was employed. Where significant *F* values were found a Bonferroni step-wise correction was employed to determine the location of variance. Two-way repeated measures Friedman's ANOVA and Wilcoxon tests follow-up tests were employed for the mood questionnaire data. Statistical significance was set at *p* < 0.05. Data are reported as mean ± SEM.

## Results

The baseline characteristics of the participants are presented in Table 1. Participants were overweight (body fat: 32.1 ± 2.1%), with a VO_2peak_ of 30.4 ± 4.8 mL.kg^−1^.min^−1^.

**Table 1:** Baseline characteristics of participants (*N* = 12).

**Variable**	**Mean ± SD**
Age, *y*	45.5 ± 2.3
Height, *cm*	164.1 ± 4.8
Body weight, *kg*	65.5 ± 8.5
BMI, *kg/m^2^*	24.6 ± 3.1
Body fat, %	32.1 ± 2.1
VO_2peak_, *mL·kg^−1^·min^−1^*	30.4 ± 4.8

**Figure 2:**
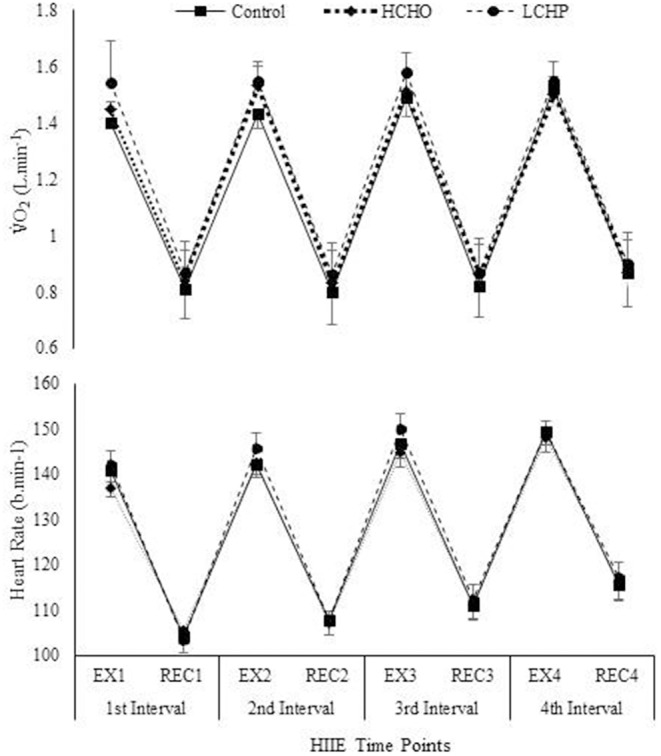
VO_2_ consumption and Heart Rate during HIIE protocol in control, HCHO and LCHP conditions. Values are means ± SEM, *n* = 12. EX1, 4-min exercise in the 1st Interval; REC1, 3-min active recovery in the 1st interval; EX2, 4-min exercise in the 2nd Interval; REC2, 3-min active recovery in the 2nd interval; EX3, 4-min exercise in the 3rd Interval; REC3, 3-min active recovery in the 3rd interval; EX4, 4-min exercise in the 4th Interval; REC4, 3-min active recovery in the 4th interval.

**Figure 3:**
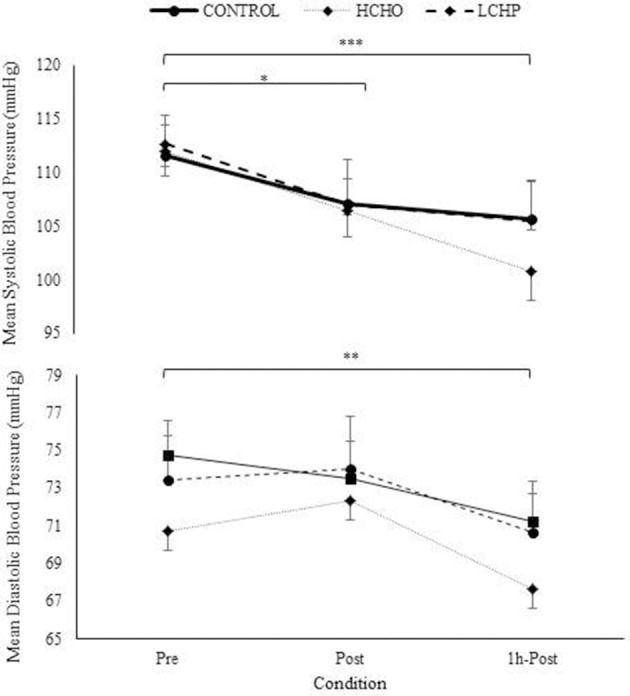
Mean, Systolic, and Diastolic Blood Pressure at pre, post and 1h- post-exercise in control, HCHO and LCHP conditions. Values are mean ± SEM, *n* = 12. ^*^p < 0.05, ^**^p < 0.01, ^***^p < 0.001 between time points.

The HIIE as expected resulted in similar responses in heart rate, VO_2_, VCO_2_, and RER between the 3 experimental conditions (*p* > 0.05) (Figure 2). No significant differences were found in RPE between the 3 conditions at the end of the HIIE protocol (14 ± 2, 13 ± 2 and 14 ± 2 for CON, HCHO, and LCHP condition, respectively, *p* > 0.05). The acute bout of HIIE alone (control condition) resulted in significant reductions in SBP immediately post-exercise (*p* < 0.05) and 1 h-post-exercise (*p* < 0.001), while MAP and DBP was only reduced 1 h-post-exercise compared to baseline (*p* < 0.01) (Figure 3). The experimental conditions had no effect on the change in MAP, SBP, and DBP, as no significant condition-by-time interactions were observed (*p* > 0.05).

A significant condition-by-time interaction was found in glucose concentrations (*p* < 0.001) but not in lactate (*p* > 0.05). At baseline, glucose concentration was not significantly different between conditions (control; 4.23 ± 0.4, HCHO; 4.44 ± 0.5 and LCHP; 4.25 ± 0.3 mmol·L^−1^, *p* > 0.05). However, during exercise glucose concentration was found to be significant higher in the control (3.87 ± 0.2 mmol·L^−1^) and LCHP condition (3.71 ± 0.5 mmol·L^−1^) compared to the HCHO (2.86 ± 0.5 mmol·L^−1^) (*p* < 0.01 and *p* < 0.01, respectively). No difference in glucose concentration was observed post-exercise between conditions (CON; 4.48 ± 0.9, HCHO; 4.11 ± 0.9 and LCHP; 4.61 ± 0.9 mmol·L^−1^, *p* > 0.05) (Figure 6).

**Figure 4:**
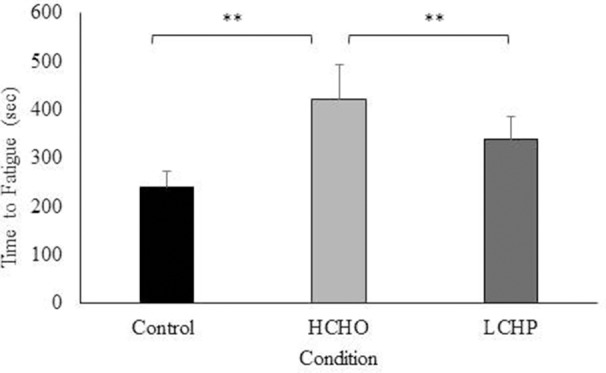
Average time in seconds to volitional cessation of exercise in control (fasted), HCHO (high carbohydrates breakfast and beverage), and LCHP (low carbohydrates-high protein- breakfast and whey protein beverage) conditions. Values are mean ± SEM, *n* = 12. ^**^p < 0.01.

In the physical performance test, time to volitional fatigue was significantly greater both in the HCHO and LCHP conditions compared to the CON (*p* < 0.01) (Figure 4). There was no statistically significant difference between HCHO and LCHP, although participants exhibited a 1.22-fold increase in time to volitional fatigue in the HCHO compared to the LCHP condition (*p* > 0.05). No significant differences were observed between the 3 conditions in HRmax (control: 169 ± 10; HCHO: 169 ± 10; LCHP: 173 ± 8 bpm) and RPE (control: 19 ± 1; HCHO: 19 ± 1; LCHP; 18 ± 2, *p* > 0.05) at the end of the physical performance test.

No differences were found in the Stroop, Shift Stroop, and three-Back cognitive function test results between conditions at baseline (*p* > 0.05) (Table 2). The acute bout of HIIE led to significant reductions in reaction time in all three experimental conditions both in the Stroop and Shift Stroop test (*p* < 0.05 and *p* < 0.001, respectively) with no difference between conditions (*p* > 0.05). Reaction time in the three-Back test and response accuracy was not affected by either HIIE or condition (*p* > 0.05).

**Table 2:** Cognitive function results.

	**Control**		**HCHO**		**LCHP**	
**Cognitive function tests**	**Pre**	**Post**	**Pre**	**Post**	**Pre**	**Post**
**STROOP TEST**						
Response accuracy	0.89 ± 0.09	0.90 ± 0.08	0.91 ± 0.07	0.90 ± 0.17	0.84 ± 0.20	0.87 ± 0.13
Reaction time (ms)	870 ± 45	850 ± 39^*^	840 ± 36	806 ± 32^*^	862 ± 40	833 ± 43^*^
**SHIFT STROOP TEST**						
Response accuracy	0.91 ± 0.06	0.94 ± 0.04	0.94 ± 0.06	0.94 ± 0.04	0.93 ± 0.04	0.93 ± 0.04
Reaction time (ms)	1,660 ± 11	1,408 ± 56^**^	1,433 ± 86	1,303 ± 80^**^	1,508 ± 90	1,295 ± 68^**^
**3—BACK TEST**						
Response accuracy	0.11 ± 0.02	0.13 ± 0.03	0.12 ± 0.03	0.13 ± 0.02	0.11 ± 0.04	0.19 ± 0.21
Reaction time (ms)	824 ± 34	783 ± 27	813 ± 27	777 ± 24	827 ± 43	763 ± 28

*Values are the average recording before (pre) and after (post) HIIE protocol. Values are mean ± SEM, n = 12*.

* *p < 0.05 between pre and post exercise*,

** *p < 0.001 between pre and post-exercise*.

**Figure 5:**
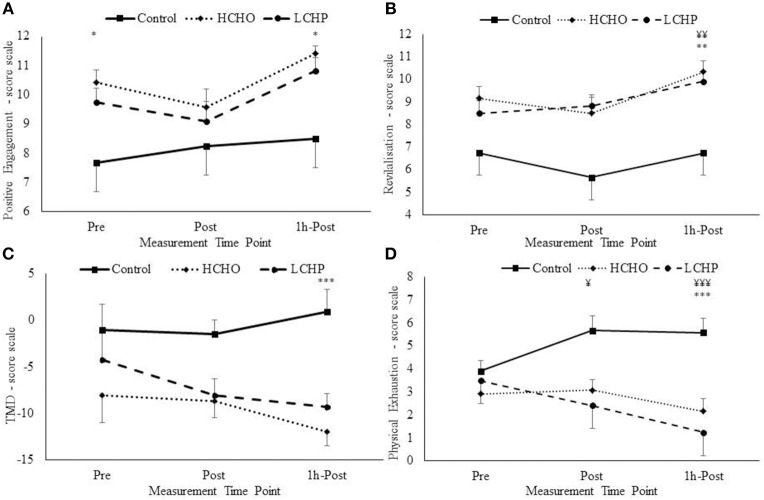
Positive engagement **(A)**, Revitalisation **(B)**, Total Mood Disturbances (TMD) **(C)**, and Physical exhaustion **(D)** changes pre, post and 1 h-post-exercise from EIFI questionnaire for HCHO, LCHP, and control. ^*^p < 0.05, ^**^p < 0.01, and ^***^p < 0.001 between HCHO and control condition, and ¥*p* < 0.05, ¥¥*p* < 0.01, ¥¥¥*p* < 0.001 between LCHO and control condition. Values are mean ± SEM, *n* = 12.

At baseline, only the HCHO condition resulted in significant improvements in mood as evident by increases in positive engagement, compared to CON and LCHP (*p* < 0.05), with no differences in any of the other mood measures (Figure 5).

**Figure 6:**
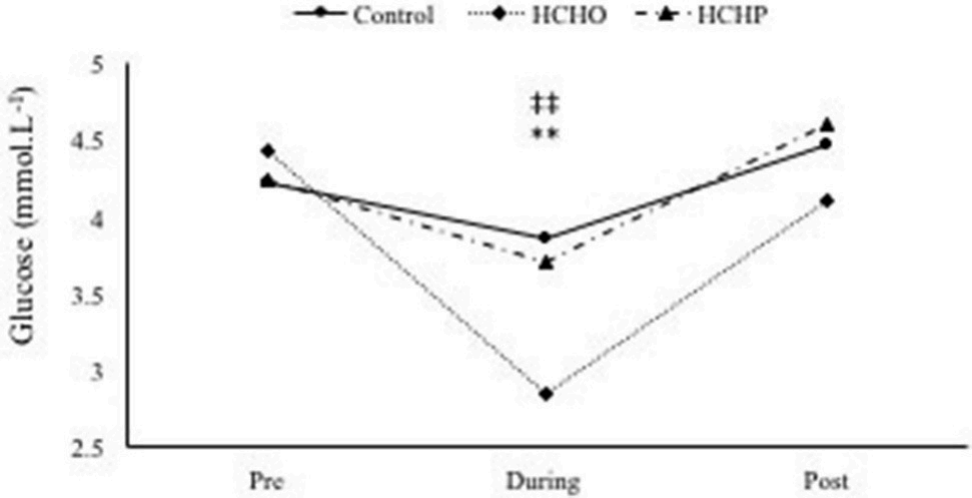
Glucose concentration during HIIE protocol in control, HCHO and LCHP conditions. ^**^p < 0.01 between HCHO and control condition, and ‡‡*p* < 0.01 between HCHO and LCHO condition. Values are means ± SEM, *n* = 12.

Post-exercise, the acute bout of HIIE alone (control condition) did not affect mood as seen by the lack of changes in positive engagement, revitalization, tranquility, and physical exhaustion immediately post and 1-h post-exercise (*p* > 0.05). However, significant condition-by-time interactions were revealed in positive engagement, revitalization, physical exhaustion, and total mood disturbance (TMD) (Figure 5). Specifically, immediately post-exercise physical exhaustion was significantly decreased only in the LCHP condition compared to CON (*p* < 0.05), with no differences found with the HCHO condition (*p* > 0.05). At 1-h post-exercise significant differences were found in mood between the three conditions, with only the HCHO condition showing significant improvements in positive engagement and TMD compared to CON and LCHP (*p* < 0.05). In addition, 1-h post-exercise both HCHO and LCHP conditions resulted in significant improvements in revitalization (*p* < 0.01) and physical exhaustion (*p* < 0.001) compared to the CON condition.

## Discussion

The findings of the present study demonstrate for the first time that a single bout of high intensity interval exercise is effective in inducing improvements in cardiovascular risk factors such as blood pressure and cognitive function, but has no effect on the mood of overweight perimenopausal women. Furthermore, it provides new scientific evidence on the importance of pre-exercise carbohydrate feedings on mood disturbance and positive engagement to exercise in overweight perimenopausal women, two exercise adherence factors of paramount importance for this population.

A novel finding of the present study is that interval exercise alone led to significant improvements both in systolic and diastolic blood pressure post-exercise in our overweight perimenopausal women and adds new information to the existing literature on the acute hypotensive effects of continuous exercise in older adults. To the authors' knowledge no other studies have been conducted investigating the acute effects of interval exercise on the blood pressure of this high risk population. Moreover, no other studies have assessed the acute effects of the specific clinical protocol of intermittent exercise used in the present study in older perimenopausal woman, an exercise protocol that has been shown to be advantageous in terms of exercise and health adaptations in older and clinical populations (13). Our findings are in agreement with a limited number of studies investigating the acute effects of HIIE on the blood pressure of older individuals. Specifically, an acute bout of HIIE has been reported to cause greater reductions in systolic blood pressure compared to 20 min moderate intensity continuous exercise in elderly hypertensive women aged ≥ 65 years, as well as to reduce ambulatory daytime diastolic blood pressure and arterial stiffness in older hypertensive males and females (20, 21). Interval training for 12 weeks has also been reported to lead to significant reductions in blood pressure and improvements in total peripheral resistance and flow mediated dilatation in hypertensive patients, while no improvements have been noted with continuous exercise training (22). The reported advantageous effects of HIIE on blood pressure in the present study are promising and strengthen the existing evidence on the efficacy of interval exercise as a therapeutic preventive exercise modality for hypertension. Nonetheless, as the present study has investigated older women that are not hypertensive, it may attenuate the relevance to clinical populations. Moreover, as the present study has only examined the acute effect of exercise, future research is needed to establish whether there is a chronic effect of interval exercise in the cardiovascular health of pre- and post- menopausal women.

Interval exercise alone did not affect the mood state of our perimenopausal women. Our findings are in disagreement with studies in adults demonstrating improvements in mood and state anxiety with continuous exercise (23), and only one published study on interval exercise reporting increased pleasure and enjoyment of exercise in older adults (24). However, similar to our results, 2 weeks of HIIE training have been found ineffective to enhance mood in both male and female adults (11). A possible reason for the lack of mood improvements in the present study is the lower overall volume of exercise as well as the timing of mood assessment. Previous studies have shown significant enhancements in mood only 1 h after the cessation of exercise but not immediately post-exercise in adults (23, 25). It has been speculated that adaptations in mood take longer to be evident after exercise, potentially due to the confounding effect of tiredness that people experience immediately after exercise (23, 25). Despite the lack of mood changes, interval exercise alone did affect the cognitive capacity of our older women, as evident by reductions in reaction time post-exercise. There is a lack of research evidence on the acute effects of interval training on cognition in older populations. A recent study investigating 3 different exercise training modalities for 16 weeks in older adults found greater cognitive improvements in the interval exercise group vs. the aerobic or resistance training group (26). In continuous exercise, extensive research on both humans and animals has demonstrated beneficial effects of exercise on cognitive and brain function and protection against the development of neurodegenerative diseases, especially later in life (27). Recently, Chang et al. (28) showed that a single bout of resistance exercise enhanced cognitive performance across all Stroop conditions in adults. Hence, due to the limited evidence on interval exercise and cognition in this population of older women, only hypotheses can be stated at this point and further research needs to investigate whether interval training can be beneficial for cognition in older populations.

The second aim of the present study was to assess whether the pre-exercise availability of carbohydrates vs. protein, would affect differently the psychophysiological responses to HIIE in perimenopausal women. We found that both pre-exercise feedings improved exercise tolerance, with trends however suggesting greater exercise tolerance improvements by 1.22-fold with the HCHO vs. the HPLC feedings. Our results are similar with studies in athletes showing improvements in exercise tolerance with greater carbohydrate availability through dietary supplementation vs. placebo (29, 30). However, only one study has been published comparing high carbohydrate vs. high protein pre-exercise meals on exercise tolerance and has found similar greater improvements in exercise performance with the carbohydrate vs. the protein feedings in young adults (31). At present, due to the lack of previous evidence in older women, we cannot make definite conclusions. Nevertheless, the present findings might indicate that high carbohydrate pre-exercise feedings could be advantageous for exercise tolerance in this population compared to high protein feedings. It is established in the literature that reduced carbohydrate availability pre-exercise, as in the case of our high protein pre-exercise feeding, can have limiting effects on glucose availability and uptake in the brain and the active musculature leading to central and peripheral fatigue, respectively (27). Moreover, low carbohydrate availability in the breakfast meal pre-exercise can impair the replenishment of liver and muscle glycogen stores after an overnight fast and hence exercise capacity (32).

Greater improvements in the total mood disturbance score and positive engagement with exercise were found with the high carbohydrate vs. the high protein pre-exercise feedings in our female population; while both diets induced improvements in physical exhaustion and revitalization compared to the control fasting condition, possibly due to the overall effect of feeding. The greater enhancements in total mood disturbances seen in our study with the high carbohydrate diet compared to the high protein diet are in agreement with previous findings in endurance trained runners (33) and women with premenstrual syndrome (34) and have been attributed to the greater blood glucose availability and, respectively, the increased serotonin synthesis and secretion in the brain (35). No previous studies have investigated the effects of high protein vs. high carbohydrate pre-exercise feedings on mood and feelings of engagement and enjoyment of exercise in older women after HIIE. The findings of the present study potentially shed some light on the possible negative effects that high protein diets might have on the overall mood and positive engagement with exercise in older populations. Taken into account that exercise adherence in these population relies heavily on the exercise being enjoyable, engaging and improving overall mood, it is imperative that more research is conducted to establish whether high protein diets can adversely alter the beneficial effects of exercise on mood. However, since the high protein diet led to similar improvements to the high carbohydrate diet in two components of the POMS test, the revitalization and physical exhaustion feelings post-exercise, it warrants caution in the interpretation of the present data and demonstrates the importance of more research studies to be conducted in this population.

Although the findings of the present study provide some novel important insights on the effectiveness of a single bout of interval exercise with low and high carbohydrate availability on older women in exercise performance, cognition and mood, there are important limitations that need to be acknowledged. The acute nature of the present study is limited in its capacity to extrapolate its findings and provide any answers on the long-terms effects of interval exercise with different nutritional interventions in older women. Moreover, although we designed a randomized cross-over study, there is a small possibility of an order-effect to take place in our findings. Finally, the low number of participants might have affected the ecological validity of our findings; a larger number of participants might had provided more insights on mood and cognitive adaptations observed with the interventions. Hence, the findings of the present study need to be interpreted with caution and future research studies need to be conducted in a larger population sample of perimenopausal women to investigate the chronic effects of such interventions on mood, cognition, and exercise engagement.

## Conclusions

The present study demonstrates that a single bout of high intensity interval exercise can improve blood pressure and cognitive function in overweight perimenopausal women. Moreover, if this type of exercise is supported by high carbohydrate pre-exercise feedings such as the typically prescribed high carbohydrate breakfast 2–3 h prior to exercise and a carbohydrate sports drink immediately prior to exercise, it can lead to improved mood, exercise engagement and possibly greater exercise tolerance. These findings potentially demonstrate the clinical importance of carbohydrates as a pre-exercise meal for this population. Moreover, it raises questions on the use of high protein, low carbohydrate diets for perimenopausal women especially when they're engaged with regular exercise training. It needs to be taken into consideration that such protein diets might limit the beneficial effects of exercise on mood, exercise enjoyment and exercise tolerance in overweight perimenopausal women. Future studies should investigate the chronic effects of interval exercise in combination with different nutritional interventions on the exercise capacity, mood and cognitive function of older perimenopausal women.

## Author Contributions

IG participated in the concept and design of the experiment, the analysis and interpretation of the data, the contribution of reagents, materials and analysis tools, and the writing of the paper. MK-N participated in the design of the experiment, the performance of the experiments, the analysis and interpretation of the data, and the writing of the paper. EW participated in the analysis and interpretation of the data, and the writing of the paper.

### Conflict of Interest Statement

The authors declare that the research was conducted in the absence of any commercial or financial relationships that could be construed as a potential conflict of interest.
